# Hallucinations: A Functional Network Model of How Sensory Representations Become Selected for Conscious Awareness in Schizophrenia

**DOI:** 10.3389/fnins.2021.733038

**Published:** 2021-11-23

**Authors:** Stephanie M. Hare

**Affiliations:** Department of Psychiatry, Maryland Psychiatric Research Center, University of Maryland School of Medicine, Baltimore, MD, United States

**Keywords:** hallucination, consciousness, global workspace, schizophrenia, salience network, fMRI

## Abstract

Hallucinations are *conscious* perception-like experiences that are a common symptom of schizophrenia spectrum disorders (SSD). Current neuroscience evidence suggests several brain areas are involved in the generation of hallucinations including the sensory cortex, insula, putamen, and hippocampus. But how does activity in these regions give rise to aberrant *conscious* perceptions that seemingly invade ongoing *conscious* experience? Most existing models assume that sensory representations are sometimes spontaneously activated in the brain, and that these spontaneous activations somehow play a causal role in the generation of hallucinations. Yet, it remains unclear how these representations become *selected* for conscious processing. No existing theory of hallucinations has specified such a “selection mechanism.” Global Workspace (GW) theorists argue that the brain’s interconnected processors select relevant piece(s) of information for broadcasting to other brain processors, rendering the information accessible to consciousness; this process known as “ignition” is associated with synchronized activity across distributed cortical and subcortical brain regions. Yet, it remains unclear how certain information and representations become selected for conscious processing. While GW theorists maintain that attention plays an important role, they have not delineated a formal “selection mechanism.” This paper specifies a selection mechanism based upon two central hypotheses: (1) a functional network called the “salience network” plays a critical role in selecting sensory representations for conscious broadcast to the GW in normal (healthy) perception; (2) sensory representations become abnormally *selected* for conscious broadcast to the GW (instead of being filtered out of consciousness) in individuals with SSD that experience hallucinations.

## Introduction

Hallucinations are a common symptom of schizophrenia spectrum disorders (SSD), although they are reported to a lesser degree by those with other psychiatric conditions, the general healthy population, and can result from prolonged alcohol and drug misuse (McCarthy-Jones, 2012). Auditory hallucinations are the most common type of hallucination in SSD with roughly 60–80% of individuals with SSD reporting hearing voices (e.g., auditory verbal hallucinations) or other sounds that nobody else hears (Sartorius et al., 1986; McCarthy-Jones, 2012; Waters et al., 2014). Hallucinations can be defined as “perception-like experiences with the clarity and impact of a true perception but without the external stimulation of the relevant sensory organ (American Psychiatric Association, 2013, p. 822).” This definition highlights that hallucinations share many of the same phenomenological features as “true perceptions,” but the key point of divergence lies in the source of the event: true perceptions are caused by external events impressing their features on relevant sensory organs, while hallucinations are not caused by an external source. What then causes hallucinations in individuals with SSD? This question lacks a straightforward answer as there are many theoretical models of hallucinations.

In recent years, predictive processing models have arguably become the dominant approach to explain hallucinations in SSD. There are many subtypes of predictive processing models that include self-monitoring approaches (Farrer and Franck, 2007; Ford and Hoffman, 2013) and Bayesian approaches (Friston, 2005; Siemerkus et al., 2019). Both approaches maintain that a critical function of our brains is to make accurate predictions; to do this, our brains are on a quest to minimize prediction errors (e.g., mismatches between outcomes we anticipate vs. outcomes we actually perceive). Our brains rely on models we develop over time including models of the sensory consequences of our actions (e.g., the sensations I expect when I move my body this way, or the expectation of hearing my voice when I begin to speak), models of social interactions (e.g., how I expect someone to respond in a particular way when I say or do something) or models of our environment (e.g., how I expect it to be raining when I step outside based on today’s meteorology report). Self-monitoring theories emphasize the predictions that we make about the sensory consequences of our motor actions (including speech), and refer to this predictive signal as a *corollary discharge* (Ford et al., 2001; Ford and Mathalon, 2005, 2019). Meanwhile, Bayesian approaches offer a more general framework for thinking about how our prior beliefs about what will happen next (referred to as “priors”) guide our inferences in noisy or ambiguous environments (Friston, 2005; Siemerkus et al., 2019). A central idea in Bayesian models of perception is that the initial prior is integrated and compared with new perceptual information conveyed by the sensory organs to produce the final percept (referred to as a “posterior”) (Friston, 2005; Siemerkus et al., 2019). In both self-monitoring and Bayesian theories, if there is a mismatch between the prediction (e.g., “corollary discharge” or “prior”) and the incoming information conveyed from the sensory organs to the associated brain sensory pathways, then a prediction error occurs.

Most existing theories of hallucinations converge on the assumption that sensory representations are sometimes spontaneously activated in the brain, and that these spontaneous activations somehow give rise to hallucinations (Ford et al., 2009; Cho and Wu, 2014). Self-monitoring theories emphasize the contribution of failures of the brain’s systems for making predictions of the sensory consequences of our actions, maintaining that the brain becomes biased or *tuned in* to process internally generated sensory signals whenever they are spontaneously emitted (Ford et al., 2009), while other types of predictive processing approaches argue that patients with SSD have a prediction error deficit, which begets resting hyperactivity of sensory cortex leading to hallucinations (Horga et al., 2014).

Despite these strengths of predictive processing theories to explain certain aspects of hallucinations in SSD, current theories of hallucinations have many shortcomings. First, while spontaneous-activation theories account well for the empirical finding that hallucinations are often associated with abnormal activity in primary and association sensory cortices both during rest (Gavrilescu et al., 2010; Hoffman et al., 2012; Sommer et al., 2012; Shinn et al., 2013; Clos et al., 2014), task performance (Horga et al., 2014; Powers et al., 2017) and the active state or experience of hallucinations (Jardri et al., 2011) in individuals with SSD, these theories fail to account for empirical findings suggesting the interplay of distributed regions beyond the sensory cortex that seem to play an important role in the generation of hallucinations in SSD including the inferior frontal gyrus (Hoffman et al., 2012; Shinn et al., 2013), insula (Sommer et al., 2012; Clos et al., 2014), putamen (Hoffman et al., 2012), and hippocampus (Sommer et al., 2012; Clos et al., 2014).

In addition, current models of hallucinations (and normal perception for that matter) fail to account for how spontaneously activated sensory representations become selected for higher conscious processing. For instance, it has been shown that the speech-sensitive region of auditory cortex spontaneously activates during periods of silence in healthy adults, and yet these individuals did not report hearing voices (Hunter et al., 2006). Consistent with the reported finding of elevated cortico-striatal connectivity in individuals with SSD that hear voices (Hoffman et al., 2012), Ford and Hoffman (2013) speculated that the threshold for consciousness of sensory representations may be reduced in individuals with SSD that hear voices (Ford and Hoffman, 2013), but the authors provide no further theoretical or empirical evidence to support this claim. This paper addresses these gaps and first brings to light recent theoretical and empirical work supporting the hypothesis that a functional brain network called the “salience network” plays a critical role in selecting and gating sensory representations for higher conscious processing in normal (healthy) perception. Next, the paper puts forth the novel hypothesis that sensory representations become abnormally *selected* for conscious processing (instead of being filtered out of consciousness) in individuals with SSD that experience hallucinations.

## Conscious Broadcasting: The Global Neuronal Workspace

Despite limits in fully explaining the neural origins (or correlates) of consciousness, advances in neuroscience have provided valuable insights into the brain’s organization including the existence of distributed modules that specialize in processing particular types of information, often at an unconscious level. But, how does informational exchange occur between these specialized (unconscious) processors to facilitate complex problem-solving?

Baars (1988) originally proposed that informational exchange is facilitated by a global workspace (GW) that consciously broadcasts information globally to the “audience” of specialized brain modules so they can interpret and understand the message. Given that only a very small amount of processed information in the brain seems to enter our limited stream of consciousness at any given moment, Baars develops a cognitive theory in which consciousness is closely tied to this GW system such that we only become consciously aware of processed information and representations when they are broadcast to the GW.

Baars (1988, 2019) also develops a rudimentary neurobiological theory of a *global neuronal workspace*, which specifies the brain “hardware” and spatiotemporal dynamics that support conscious broadcast of selective pieces of information. He emphasizes the role of the cortico-thalamic-reticular activating system, which has core hubs in the brainstem reticular formation and thalamus with efferent connections spouting up to the cortex with a “fountain-like” structure (Arguinchona and Tadi, 2021). Further, he claims that the structure of this system allows for sampling inputs from specialized modules all over the brain and broadcasting relevant pieces of information globally via bidirectional, long-distance cortico-thalamic projections (Baars, 1988). In a recent update to his GW theory, Baars speculated that the hippocampus may play a role in specifying conscious experiences (Baars, 2019), but did not specify how hippocampal signaling and/or functional interactions with other neural subsystems might give rise to conscious experiences.

More recent proponents of GW theory have expanded upon Baars’ core ideas, emphasizing the role of lateral fronto-parietal networks in conscious perception (Dehaene et al., 2003; Dehaene and Changeux, 2011) and developing the notion of *ignition*, which is “characterized by the sudden, coherent, and exclusive activation of a subset of neurons coding the GW for the current conscious content (Mashour et al., 2020).” Despite these divergences from Baars’ theory, the core idea of GW theory remains: information must spread throughout the brain to distributed cortical and subcortical regions, and widespread (vs. local) activity is associated with broadcasting information to consciousness.

Given that only a very small amount of information processed in the brain enters our stream of consciousness at any moment, an important question arises: how do brain systems determine or regulate which pieces of information should be consciously broadcast to the GW? Baars claims that there are many input streams that could be consciously broadcast at any given moment, but that a single input becomes broadcast in a “winner-take-all” fashion (Baars, 2019). Moreover, he argues that both automatic attention (e.g., reflexively orienting our attention to personally significant stimuli such as our own name) and voluntary attention (e.g., conscious deliberation amongst a variety of options) play a role in assigning priority to potentially conscious events (Baars, 1988), with attention acting as a spotlight that selects vital information (Baars, 1997). Beyond this, Baars does not specify how relevant information becomes selected for conscious broadcasting to the GW. Recent proponents of GW theory have argued that attention may act as a set of filters (operating consciously or unconsciously), with the final one gating entry into the GW of consciousness (Mashour et al., 2020).

While attention seems to play a role in specifying which pieces of information become selected for higher conscious processing (and broadcast to the GW), it remains unclear how certain information and representations become selected for higher conscious processing and entry into the GW. Beyond speculating that attention plays some role in this process, key pioneers in the field of GW—Baars and Dehaene—have not yet specified details of a “selection mechanism.” In the following section, I bring to light recent theoretical and empirical work that has not gained strong traction in the field of GW theory, which supports the idea that the brain may have a specialized functional network that serves as the gatekeeper of the GW.

## The Salience Network: Gatekeeper of the Global Workspace

Advances in human neuroimaging have provided insight into how the brain is organized into functional networks (e.g., anatomically distributed brain regions that show consistent patterns of functional co-activation and appear to work together to facilitate a particular function). In the early 2000s, two core functional networks were discovered that demonstrated antagonistic activity patterns (e.g., when one network was active, the other network was inactive, and vice versa). The default-mode network (DMN) was consistently active during periods of undirected internal thoughts or mind-wandering, and its core hubs spanned anterior (medial frontal) and posterior (cingulate and precuneus) midline structures (Raichle et al., 2001; Fox et al., 2005; Raichle, 2015). An executive control network (ECN) contained core hubs in lateral fronto-parietal regions and was consistently active during tasks that required externally focused attention (Fox et al., 2005; Seeley et al., 2007). Notably, the ECN bears much similarity to the lateral fronto-parietal networks associated with conscious perception described by recent GW theorists (Mashour et al., 2020).

It is thought that DMN/ECN antagonistic activity may be important for flexible switching between periods of externally oriented attention fixed on a particular task or goal and periods of internally directed, stimulus-independent thought (Seeley et al., 2007; Menon and Uddin, 2010; Menon, 2011, 2015). A third network was also discovered whose activation was observed across a range of conditions including when participants heard an odd tone in a string of background tones, when their blood pressure changed, or when they made an error on a task (Seeley et al., 2007; Menon and Uddin, 2010; Menon, 2011, 2015). Based on this activation profile, it was thought that this network must play an important role in monitoring the relevance or *salience* of internal and external stimuli; it was coined the *salience network* (SN).

The core cortical hubs of the SN are the anterior insular cortex and the dorsal anterior cingulate cortex (Seeley et al., 2007; Peters et al., 2016). The insula’s vast structural connectivity with brain centers involved in sensory processing (Mesulam and Mufson, 1982; Mufson and Mesulam, 1982), threat (with input from amygdala) (Mesulam and Mufson, 1982; Mufson and Mesulam, 1982), and bodily homeostasis and interoception (with input from the hypothalamus and other structures) (Craig, 2002) equip it to integrate external information from one’s environment as well as information about one’s inner states (e.g., thoughts, memories, states of one’s own body, etc.) (Craig, 2002, 2009). Convergent evidence from functional MRI studies of effective connectivity using Granger Causality and dynamic causal modeling methods (Sridharan et al., 2008; Goulden et al., 2014) as well as studies of traumatic brain injury (Bonnelle et al., 2012; Jilka et al., 2014) provide compelling support that the anterior insular hub of the SN may act as a switch between internally directed and externally directed network states.

**FIGURE 1:**
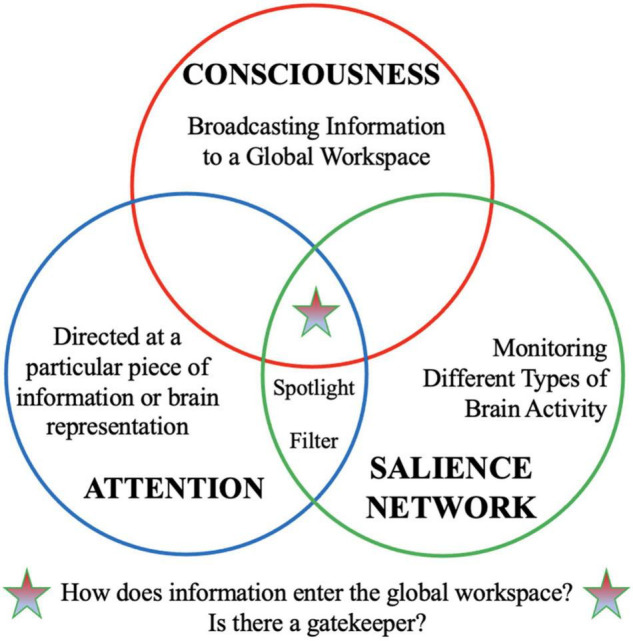
Conceptual overlap between “attention,” “consciousness,” and “salience network.” The salience network is a functional network that is thought to play an important role in filtering important information from “noise” and acts as the brain’s spotlight to tune into important information. We use similar analogies of a “filter” and “spotlight” to describe attentional processes. Meanwhile, “consciousness” is associated with broadcasting information widely to global brain workspace. This conceptual analysis raises interesting questions: How does information enters the global workspace for broadcasting to consciousness? Is there a gatekeeper that regulates information entering the global workspace?

Together, these findings suggest that the SN—and anterior insula in particular—may help filter important signal from noise and focus the spotlight on important signal(s) so that we can flexibly direct our attention to the most pressing matters. As discussed in Section 2, the analogies of a “filter” or a “spotlight” have been used to describe attention. Thus, core functions of the SN have shared conceptual overlap with the functions we often ascribe to attention (Figure 1). It has been proposed that attentional filters gate entry into the GW of consciousness (Mashour et al., 2020), but might the SN play a similar functional role?

Emerging evidence suggests that the SN—and anterior insula in particular—may play a critical role in gating or filtering information into the GW for higher conscious processing. Direct intracranial stimulation of the anterior insula (and adjacent claustrum) was shown to drastically alter the state of consciousness in a case study (Koubeissi et al., 2014), and also reliably induced strange conscious experiences (e.g., tingling, hot flashes) in other studies (Mazzola et al., 2017; Bickel and Parvizi, 2019). In addition, a slow anesthesia induction study showed that loss of conscious responsiveness was associated with suppression of anterior insular activity and disruption in frontoparietal ECN activity (Warnaby et al., 2016). A recent study of propofol titration in healthy participants similarly reported loss of conscious responsiveness was associated with dysfunction of anterior insular activity and impaired dynamic transitions between DMN and an executive attention network (Huang et al., 2021). Based on these findings, it has been theorized that the anterior insula may help regulate which piece(s) of information gain access to the GW (Michel, 2017; Evrard, 2019; Huang et al., 2021) by representing the salience of different types of brain representations (e.g., of homeostatic, sensory, and emotional states) and mapping whether the representation has priority to enter the GW of consciousness (Michel, 2017).

**FIGURE 2:**
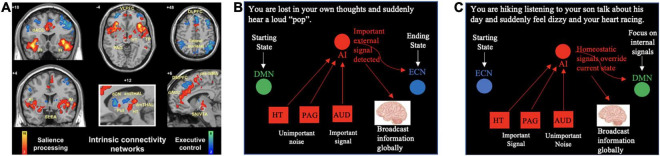
The SN monitors input from all different brain centers to respond to our shifting internal and external (environmental) states. **(A)** The first study of the “salience network” identified several subcortical hubs of the network including the amygdala (conveying information about threats and fear responses), the hypothalamus (conveying information about bodily homeostasis), and the periaqueductal gray (conveying information about pain). Adapted from Seeley et al. (2007) (Copyright 2007, Society for Neuroscience). **(B,C)** Depict two different scenarios in which the salience network monitors incoming information and reorients our attention in response to our shifting environments or shifting thoughts. AI, anterior insula; antTHAL, anterior thalamus; AUD, auditory processing centers; ECN, executive control network; dCN, dorsal caudate nucleus; DMN, default mode network; dmTHAL, dorsomedial thalamus; DMPFC, dorsomedial prefrontal cortex; HT, hypothalamus; PAG, periaqueductal gray; Put, putamen; SLEA, sublenticular extended amygdala; SN/VTA, substantia nigra/ventral tegmental area; TP, temporal pole; VLPFC, ventrolateral prefrontal cortex.

Notably, this intriguing theory has not yet gained strong traction and representation in the recent updates to GW theory by its core proponents (Baars, 2019; Mashour et al., 2020). Thus, one of the goals of this paper is to bring light to this theory that has been somewhat underrepresented in the existing literature on the GW theory. My second goal is to extend this theory, by developing a functional network account of how different hubs of the SN – not just the anterior insula – work together to serve a gatekeeping function for higher conscious processing (and entry into the GW) (Figure 2).

In addition to its two core cortical hubs (e.g., anterior insula and dorsal anterior cingulate cortex), the SN contains subcortical hubs including subregions of the amygdala, hypothalamus, periaqueductal gray, dorsal striatum, and ventral tegmental area extending into the substantial nigra (Seeley et al., 2007; Peters et al., 2016; Figure 2A). It has been proposed that the anterior insular cortex receives input from each of these structures and continuously monitors for salient information about our surroundings (conveyed by primary and associative sensory cortices), threats (conveyed by amygdala), bodily homeostasis (conveyed by the hypothalamus), and pain and negative prediction errors (conveyed by the periaqueductal gray) (Menon and Uddin, 2010; Menon, 2015). In this way, cortical SN hubs such as the anterior insular cortex can then selectively filter “important signal” from “unimportant noise” that is being conveyed by specialized brain centers for higher conscious processing.

These functional and structural connections allow the SN to continuously monitor different types of information and detect unexpected changes from the *status quo*. A certain level of baseline “resting” activation of the SN may sustain continuous monitoring, but large elevations in SN activity beyond baseline are observed specifically during critical *shifts* and/or unanticipated *changes* in our bodies, thoughts and environments: when participants hear an odd tone in a string of background tones, when their blood pressure changes, or when they make an error on a task (Seeley et al., 2007; Menon and Uddin, 2010; Menon, 2015). The SN allows us to efficiently respond to these *changes* from the *status quo* by making use of its functional connections with the DMN and ECN to consciously broadcast important information and orient our attention to appropriate stimuli given our shifting environments and goals (externally in the cases of emerging outward threats or goals, and internally in the case of emerging shifts in thoughts or bodily homeostasis).

Figure 2 provides a framework for thinking about how the SN might monitor different types of information and proposes two scenarios for how the SN might respond to rapid and unexpected changes in the *status quo*. First, consider an individual that is lost in her own thoughts, and suddenly hears a loud “pop” sound (Figure 2B). She starts out in a DMN state with her attention focused inward on her own thoughts and her own thoughts are consciously broadcast to the GW. When she hears a loud sound that may indicate a threat, the SN initiates a shift to an ECN state in which attention is focused outwardly (toward identifying the source of the sound). Her initial conscious thoughts are interrupted, and new concerns and thoughts about her environment (e.g., “What was that sound?”; “Where is it coming from?”) are broadcast to the GW (Figure 2B).

Figure 2C depicts an alternative case in which a woman is hiking with her son on a hot summer day, listening to him talk about his day, and she suddenly feels dizzy and her heart racing. She begins in an ECN state with her attention focused on the scenic environment and the conversation with her son; this conversation is consciously broadcast to her GW (Figure 2C). The SN then detects a shift in bodily homeostasis and initiates a shift to a DMN state to focus attention on herself and current bodily state. The conscious broadcast of her son’s words to the GW are interrupted, and thoughts related to herself and her well-being (e.g., “What is happening to me?”; “Why do I feel dizzy?”; “Am I ok?”) are consciously broadcast to the GW (Figure 2C).

These two hypothetical scenarios share several important features. First, in both cases, the SN utilizes its functional connections with the DMN and ECN to reorient attention to the most pressing matters. In addition, the information (e.g., feelings, thoughts, etc.) that is initially broadcast to the GW rapidly shifts in response to changes in the environment or our bodies. While it appears that the SN is somehow involved in broadcasting pertinent information to the GW for higher conscious processing, the specific functional role that the SN plays in this process remains unclear. One plausible hypothesis is that cortical SN hubs play a critical role in monitoring information about our bodily states, environmental states, and emotional states relayed by subcortical SN hubs (e.g., hypothalamus, amygdala, etc.) and sensory cortices, and these hubs then select the most pertinent information to be broadcast to the GW of consciousness. In this way, the SN acts as a sort of *gatekeeper* of the GW. In the case of normal (healthy) perception, the anterior insular cortex may help filter out the most pressing sensory percepts and representations from background noise.

In summary, given that our goals and environments are constantly changing, the SN equips us to respond flexibly and adaptively to important changes from the *status quo*. Thus, *dysfunction* of the SN is likely to have a substantial impact on behavior and might beget different psychiatric symptoms. This is discussed in the following section.

## Hallucinations: A Case of Sensory Representations Becoming Abnormally Selected for Conscious Broadcast to the Global Workspace

It has been postulated that dysfunctional interactions between the SN and both DMN and ECN may give rise to psychotic symptoms such as hallucinations (Menon, 2011, 2015; Supekar et al., 2019; Bolton et al., 2020). Given that individuals at high risk for developing psychosis have a loss of normal, time-varying interactions between the SN and both the DMN and the ECN (Bolton et al., 2020), it has even been proposed that abnormal SN engagement with the other two networks may be a neurobiological signature of psychotic symptoms (Supekar et al., 2019). However, dysfunctional activity and volume loss of the SN has also been reported in studies of substance-abuse, attention-deficit/hyperactivity, anxiety, bipolar and major depression disorders (Goodkind et al., 2015; Sha et al., 2019), suggesting that SN dysfunction may be a more general marker of a broad range of psychiatric symptoms.

Nonetheless, prior neuroimaging research provides compelling evidence that hallucinations in SSD are strongly associated with dysfunctional SN activity (Palaniyappan and Liddle, 2012; Pu et al., 2012; Manoliu et al., 2014; Lefebvre et al., 2016; Hare et al., 2018). In patients, resting connectivity strength within the right anterior insular node of the SN was strongly correlated with hallucinations severity score (Manoliu et al., 2014). Another study reported reduced functional connectivity between the cortical SN hubs (anterior insula, anterior cingulate) in patients in the early stage of schizophrenia relative to controls, and lower gray matter volume of the right insula was associated with more severe hallucinations (Pu et al., 2012). Based on these findings, it has been theorized that aberrant SN activity may result in the inappropriate assignment of salience to normal, background resting-state activity in the brain (e.g., activity that would normally be filtered out as unimportant “noise”) (Palaniyappan and Liddle, 2012). But what signals or representations are not being properly filtered by the SN in the case of hallucinations?

**FIGURE 3:**
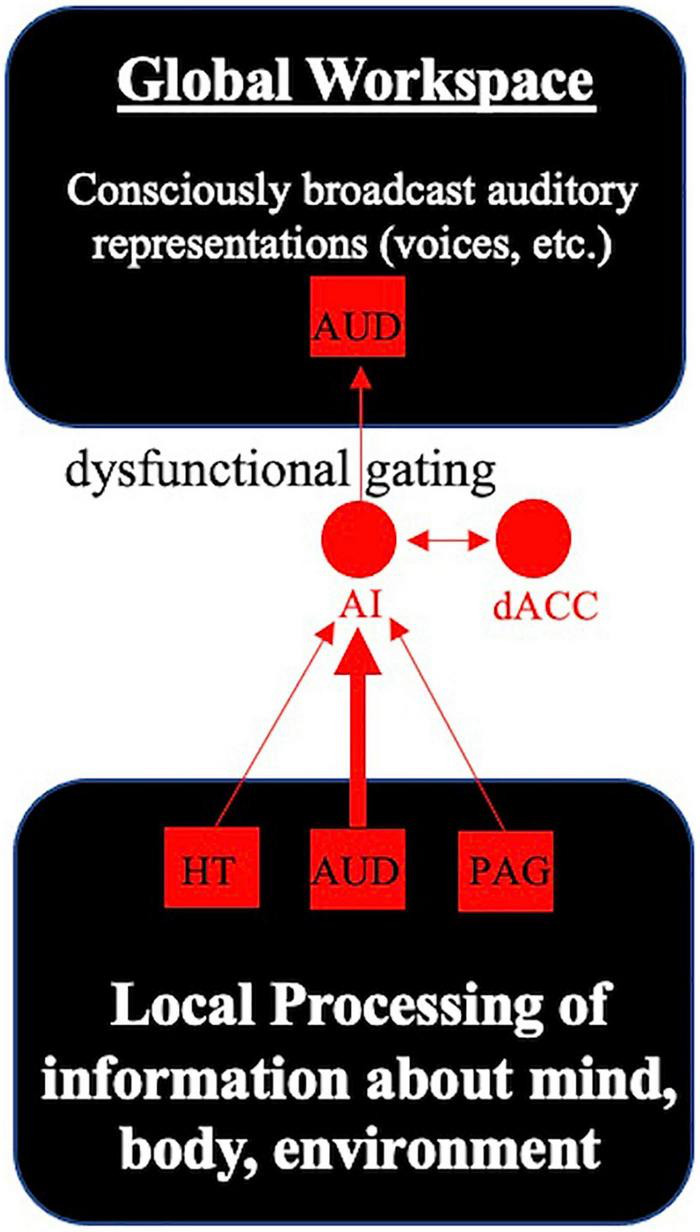
Dysfunctional gatekeeping of the global workspace leads to aberrant perceptions that invade conscious awareness. The anterior insula (AI) continuously monitors inputs from brain areas including the hypothalamus (HT), auditory cortex (AUD) and periaqueductal gray (PAG). Working with the dorsal anterior cingulate cortex (dACC), these cortical hubs of the salience network select the most relevant information (from unimportant background noise) for conscious broadcast to the global workspace. In the case of auditory hallucinations, dysfunctional gatekeeping of the global workspace by the core cortical hubs of the salience network may result in inadvertent broadcast of auditory representations (e.g., voices, sounds) to conscious awareness in some individuals with schizophrenia.

One plausible hypothesis is that the SN may not properly filter memory-based sensory representations, and this gives rise to hallucinations. A dynamic causal modeling analysis of SSD patients that probed periods of “active hallucination” vs. “no hallucination” in the scanner reported that periods of active hallucinations were associated with left hippocampal input to the SN (Lefebvre et al., 2016). Meanwhile, an analysis of resting functional connectivity between DMN, SN, sensory, and subcortical (hippocampal, striatal) networks reported that more severe auditory hallucinations in SSD patients were strongly associated with higher resting connectivity between the SN and an associative auditory network (Hare et al., 2018). Interestingly, both SSD patients reporting auditory hallucinations as a symptom and non-hallucinating SSD patients had elevated resting connectivity between the associative auditory network and the hippocampus relative to healthy controls (Hare et al., 2018). Consistent with this evidence of SN, auditory network, and hippocampal involvement in the generation of hallucinations (see also Jardri et al., 2011; Sommer et al., 2012; Clos et al., 2014; Hare et al., 2017), I hypothesize that memory-based sensory representations encoded by the hippocampus and sensory (auditory, visual) networks may not be properly filtered by the SN as “unimportant noise.” If the SN—with an important contribution of the anterior insular cortex—serves as a gatekeeper of the GW, this may result in these sensory representations being aberrantly broadcast to the GW (Figure 3), which may manifest as unbidden perception-like experiences (e.g., hearing voices in the case of aberrant selection of auditory verbal imagery or memories, having strange visions in the case of aberrant selection of visual imagery or memories, etc.).

A major strength of this functional network model is that it provides an explanation for how sensory information and representations (e.g., voices, sounds, visions) may uncontrollably invade one’s stream of consciousness in certain cases of SSD. However, there are several limitations of my theoretical model to consider. First, my model was built upon the presupposition of a GW theory of consciousness. GW theory has several strengths as a theory of consciousness, but also may have shortcomings in explaining certain phenomena relative to competing theories of consciousness (Brown et al., 2019).

Next, on my account, it is unclear whether and how functional interactions between SN hubs might actively facilitate entry of certain information available for conscious broadcast (but not other types of information) and what role(s) the other major SN hub—the dorsal anterior cingulate cortex (dACC)—may play in this process. As previously mentioned, a study by Hunter et al. (2006) reported that speech-sensitive auditory cortex spontaneously activates during periods of silence in healthy adults, and yet these individuals did not report hearing voices. During these periods of activation, the ACC was often co-active with auditory cortex; the authors speculated that the ACC may somehow modulate cortical activity (Hunter et al., 2006), but did not specify a mechanism that explains how this occurs and why these individuals did not hear voices.

The experience of hearing voices (e.g., auditory verbal hallucinations) is one of the most prevalent symptoms of SSD. Roughly 60–80% of individuals with SSD reporting hearing voices at some point in the course of the illness (Sartorius et al., 1986; McCarthy-Jones, 2012; Waters et al., 2014), although verbal hallucinations are reported to a lesser degree by those with other psychiatric conditions, the general healthy population, and can result from prolonged alcohol and drug misuse (McCarthy-Jones, 2012). One recent study of two groups of voice hearers (those with a psychiatric diagnosis of SSD and “healthy voice hearers” without a formal diagnosis that were recruited from a community of psychics) may provide further insight into the ACC’s involvement in healthy auditory perception and cases of aberrant perception and hallucinations (Powers et al., 2017). The study used Pavlovian conditioning (pairing delivery of light with a tone in a noisy environment) to induce conditioned hallucinations (hearing a tone when one was not delivered). Both groups of voice hearers were more susceptible to this conditioned hallucination effect. Notably, the “voice hearers” failed to activate ACC during correct responses (e.g., accurately identifying that no tone was delivered) relative to non-voice hearers (including an SSD control group that did not report hearing voices) (Powers et al., 2017).

Together, this work (Hunter et al., 2006; Powers et al., 2017) suggests that the ACC is playing some important role(s) in auditory perception and hallucinations, but the specific roles it is playing remain unclear. Given its connections with motor areas, the dACC is often taken to play a crucial role in motor response selection (Menon, 2011, 2015). This view aligns with basic neuroscience research suggesting that when task conditions shift such that prior motor plans must be inhibited, the ACC attenuates activity in downstream targets to inhibit the prior learned motor response (Brockett et al., 2020). These findings may shed light on the challenging question of whether and how functional interactions between SN hubs might facilitate entry of certain information available for conscious broadcast (but not other types of information). One possibility is that the anterior insula monitors activity in different brain networks for important information, and when there is a need to shift behavior in the face of changing circumstances, the insula signals to the ACC to modulate activity in downstream sensorimotor systems to inhibit previous action plans and update action(s) accordingly. In the case of healthy auditory perception, the auditory cortex may undergo periods of spontaneous activity at rest, but co-activation of ACC may signal to downstream targets (including auditory processing systems) that this is unimportant noise that should be ignored. In the case of auditory hallucinations, failure to properly engage the ACC may convey the erroneous signal that this is important information that should be broadcast to the GW (when it would normally be filtered out of conscious awareness). Given our currently limited understanding of the functional interactions between the ACC and auditory cortex during auditory perception (and the special case of hallucinations), this is admittedly speculative.

Speaking to the prevalence of auditory hallucinations in SSD, my functional model presupposes that the SN persistently makes errors in selecting *auditory representations* for conscious processing and entry into the GW. But why might the SN consistently broadcast voices or other types of auditory representations (vs. broadcasting other types of information)? One plausible hypothesis is that large fluctuations in a hyperexcitable auditory cortex consistently “flip the switch” of the SN and broadcast auditory representations to the GW. This hypothesis is consistent with empirical support for spontaneous activation theories demonstrating abnormal activity in auditory cortices at rest (Gavrilescu et al., 2010; Hoffman et al., 2012; Sommer et al., 2012; Shinn et al., 2013; Clos et al., 2014) and heightened co-activation of SN and auditory cortex at rest (Hare et al., 2018) in individuals with SSD who experience hallucinations. Alternatively, the SN might transform the input from auditory regions in some way or another interacting network might try to make sense of why the noisy input is flagged as relevant. Future research studies should explore these hypotheses that may explain why individuals with SSD are more prone to auditory (vs. other types of) hallucinations.

Finally, on my functional network account of auditory hallucinations, it remains unclear how abnormal flagging by the SN begets the experience of hearing speech from an external source. My model proposes that abnormal flagging of auditory memories by the SN results in the conscious broadcast of past words and conversations stored in memory (Figure 3), but this hypothesis may only be partially consistent with findings from the largest study of phenomenological features of auditory verbal hallucinations (Nayani and David, 1996). In the study of 100 patients with a psychotic disorder, 61% of those surveyed admitted to knowing the identity of one or more of their voices. Nayani and David (1996) argued that “hallucinated voices were often known to the patient in real life, indicating that they may be modeled on the memory of a real voice (p. 181).” While my theory is consistent with this broad claim, I maintain that my theory may only explain some instances of hallucination (e.g., those experiences that seem to be grounded in past experiences and memories) but not others (e.g., those that appear to have no connection to past experiences or memories). Despite these limitations, my functional model of hallucinations advances the field of neuropsychiatry by providing a theory that (1) is consistent with empirical evidence suggesting involvement of multiple, distributed brain areas and networks in the generation of hallucinations, and (2) explains how sensory representations may become selected for conscious broadcast in an unbidden manner.

## Discussion

In summary, current gaps in understanding the underlying neuroscience of conscious awareness and hallucinations in SSD have left several questions unanswered. Prior literature on GW theory failed to specify whether and how information gains access to the GW for conscious broadcasting (Figure 1), while prior literature on the neuroscience of hallucinations in SSD failed to account for how spontaneously activated sensory representations become involuntarily selected for higher conscious processing in individuals with SSD. This paper developed a functional network framework positing that: (1) the SN plays a critical role in selecting and gating sensory representations for higher conscious processing in normal (healthy) perception; and (2) sensory representations become abnormally *selected* for conscious processing (instead of being filtered out of consciousness) in individuals with SSD that experience hallucinations.

While this framework provides answers to several important questions, many questions remain unanswered. First, adopting a functional network account of hallucinations implicating dysfunction of the auditory cortex, hippocampus, SN and its interactions with the DMN and ECN, is there a predominate cause of hallucinations in one or more of these circuits in individuals with SSD? From a developmental perspective, does one of these systems take a “first hit” in childhood or adolescence and predispose individuals to later develop hallucinations and a psychotic disorder? These questions are an important avenue for future research.

From a basic neuroscience perspective, we might also wonder how relevance or salience is coded by respective nodes of brain networks – hypothalamus, amygdala, sensory cortex, etc.—such that a relevant piece of information “flips the switch of the SN” and is selected for higher conscious processing. Is there a common code or different codes for each brain station? Overall, we know very little about how the brain codes and interprets “important signal” (that merits further conscious processing) from noise. One factor that may influence this is signal amplitude (e.g., how large is the fluctuation in activity from the background/resting activity), but, how might the timing of different brain signals come into play? The particular frequency (e.g., alpha, beta, gamma) of signals and/or their timing (e.g., are the signals locked to a particular phase of the ongoing frequency) may be critically important. These questions should also be addressed in future research studies.

In summary, my functional network model (Figure 2), provides a framework for thinking about how our brains continuously monitor information (including sensory information and representations), detect important or unexpected changes from the *status quo*, route that information for higher conscious processing and re-orient our attention to the most pressing matters. The triple-network model also helped generate the novel prediction that hallucinations in SSD may arise due to sensory representations becoming abnormally *selected* for conscious processing (instead of being filtered out of consciousness) by the SN. In this triple-network model, the SN acts as the lynchpin that directs activity of two anti-correlated networks: the DMN and the ECN. Returning to the idea that there is a great deal of conceptual overlap between the notions of “consciousness” and “attention” (Figure 1), two alternative hypotheses can be proposed regarding SN interactions with the DMN and ECN; the SN performs its functional roles by either (1) directing the activity of *two attention networks*—one associated with internally directed attention (DMN) and the other associated with externally directed attention (ECN)—or (2) determining which pieces of information get broadcast to *two different global neuronal workspaces*—one workspace for broadcast of internally generated information and representations relating to oneself (e.g., thoughts, information about bodily homeostatic state, past memories, etc.) (DMN) and another channel for broadcast of external information and goals (e.g., sensory percepts, information about social interactions and hierarchy, etc.) (ECN). The latter hypothesis would suggest that, in addition to there being a global neuronal workspace for conscious perception of external stimuli that is constituted by the vast spread of information to lateral fronto-parietal regions (Dehaene et al., 2003; Dehaene and Changeux, 2011), there may be a second global neuronal workspace for broadcasting information related one’s own thoughts, body, and actions. Cognitive neuroscientists, basic neuroscientists, and philosophers of consciousness should work together to address whether there is a way to test these two intriguing alternative hypotheses.

It is important to test these alternative hypotheses and other related hypotheses to improve our general understanding of the basic neuroscience governing how we process and integrate information, but also to improve our understanding of the psychopathology of different psychiatric and brain disorders. This theoretical paper focused on better understanding the neural underpinnings of hallucinations in SSD, and might partially address the question of how the threshold for consciousness of sensory representations may be altered in individuals with SSD that report hallucinations (Ford and Hoffman, 2013). Recently published work suggests that the threshold for conscious access and entry into the GW may be altered in individuals with a history of psychosis, reporting that an estimate of consciousness threshold correlated with integrity of long-distance white-matter tracts of the hypothesized global neuronal workspace (inferior frontal-occipital fasciculus, cingulum, and corpus callosum) (Berkovitch et al., 2021). While this finding provides preliminary evidence favoring the theory that schizophrenia is a “disorder of consciousness,” there is also cognitive neuroscience evidence (Nuechterlein and Dawson, 1984; Cornblatt et al., 1989; Sawaki et al., 2017) to support the theory that schizophrenia might be characterized as a “disorder of attention.” My functional network model of hallucinations in SSD is compatible with the view that both attentional processing and conscious access are likely altered in SSD, specifically predicting that dysfunctional SN communication with other networks will result in difficulties (1) shifting our attention to goal-relevant information from moment-to-moment, and (2) gating relevant information for higher conscious processing and entry into the GW.

## Author Contributions

SH independently developed the ideas, theories and hypotheses in this manuscript and drafted all versions of the manuscript.

## Conflict of Interest

The author declares that the research was conducted in the absence of any commercial or financial relationships that could be construed as a potential conflict of interest.

## Publisher’s Note

All claims expressed in this article are solely those of the authors and do not necessarily represent those of their affiliated organizations, or those of the publisher, the editors and the reviewers. Any product that may be evaluated in this article, or claim that may be made by its manufacturer, is not guaranteed or endorsed by the publisher.
